# Circulating tumor cells participate in the formation of microvascular invasion and impact on clinical outcomes in hepatocellular carcinoma

**DOI:** 10.3389/fgene.2023.1265866

**Published:** 2023-11-02

**Authors:** Bin Sun, Wei-Dan Ji, Wen-Chao Wang, Lei Chen, Jun-Yong Ma, Er-Jiang Tang, Mou-Bin Lin, Xiao-Feng Zhang

**Affiliations:** ^1^ Center for Clinical Research and Translational Medicine, Yangpu Hospital, Tongji University School of Medicine, Shanghai, China; ^2^ Department of Molecular Oncology, Eastern Hepatobiliary Surgical Hospital and National Center for Liver Cancer, Navy Military Medical University, Shanghai, China; ^3^ Department of General Surgery, Yangpu Hospital, Tongji University School of Medicine, Shanghai, China; ^4^ Department of Hepatic Surgery, Eastern Hepatobiliary Surgery Hospital, Navy Military Medical University, Shanghai, China

**Keywords:** circulating tumor cells, microvascular invasion, recurrence and metastasis, clinical treatment, hepatocellular carcinoma

## Abstract

Hepatocellular carcinoma (HCC) is a common malignant tumor worldwide. Although the treatment strategies have been improved in recent years, the long-term prognosis of HCC is far from satisfactory mainly due to high postoperative recurrence and metastasis rate. Vascular tumor thrombus, including microvascular invasion (MVI) and portal vein tumor thrombus (PVTT), affects the outcome of hepatectomy and liver transplantation. If vascular invasion could be found preoperatively, especially the risk of MVI, more reasonable surgical selection will be chosen to reduce the risk of postoperative recurrence and metastasis. However, there is a lack of reliable prediction methods, and the formation mechanism of MVI/PVTT is still unclear. At present, there is no study to explore the possibility of tumor thrombus formation from a single circulating tumor cell (CTC) of HCC, nor any related study to describe the possible leading role and molecular mechanism of HCC CTCs as an important component of MVI/PVTT. In this study, we review the current understanding of MVI and possible mechanisms, discuss the function of CTCs in the formation of MVI and interaction with immune cells in the circulation. In conclusion, we discuss implications for potential therapeutic targets and the prospect of clinical treatment of HCC.

## 1 Introduction

The latest epidemiological studies show that liver cancer (LC) is the third most common cause of cancer-related death (8.3%) in the world (Sung et al., 2021) with the characteristics of early detection difficult, high degree of malignancy, poor sensitivity to radiotherapy and chemotherapy, high recurrence rate and low 5-year survival rate (Nault et al., 2018; Li and Xu, 2019). Clinically, LC could be divided into hepatocellular carcinoma (HCC), cholangiocarcinoma (CC) and mixed liver cancer according to the histological type. Among them, HCC is the most common histological subtype, accounting for more than 75%–85% of primary liver cancer (Qiu et al., 2019). Hepatitis B virus (HBV) and hepatitis C virus (HCV) infection remain the most important risk factors for HCC globally, but with the widespread HBV vaccination for newborns, it will bring obvious benefits to reduce the incidence rate of HCC in the future. In addition, excessive alcohol consumption and aflatoxin contamination are also important risk factors. Unfortunately, the prevalence of metabolic risk factors, including metabolic syndrome, obesity, type II diabetes and non-alcoholic fatty liver disease (NAFLD) are becoming undeniable factors leading to the occurrence of HCC (McGlynn et al., 2021; Samant et al., 2021). At present, the commonly used imaging methods for clinical diagnosis of HCC include abdominal ultrasound, multiphase computed tomography (CT), and magnetic resonance imaging (MRI), which determine normal tissue and HCC tissue based on physiological differences such as different blood perfusion, as well as HCC staging.

HCC is a kind of multi-vascular malignant tumor. The formation of vascular tumor thrombus or vascular invasion is a common malignant biological phenomenon of HCC, which is likely to cause intrahepatic metastasis and seriously affect the prognosis of patients (Xu et al., 2019). The vascular invasion of HCC includes microvascular invasion and macrovascular invasion. Microvascular invasion (MVI), also known as microvascular tumor thrombus, mainly refers to the presence of cancer cell nests (the number of cancer cells ≥50) in the vascular lumen lined by endothelial cells under the microscope. As a special intermediate link in the development of HCC, MVI could further develop into macrovascular invasion (defined as invasion of tumor into a major vessel) or portal vein tumor thrombus (PVTT) (Sumie et al., 2014a; Sheng et al., 2020).

As an indicator of tumor invasiveness, MVI is one of the risk factors leading to tumor recurrence in HCC, which seriously affects the long-term prognosis of patients (Song et al., 2018; Qi et al., 2019). At present, the clinical diagnosis of MVI still depends on the pathological evaluation of HCC after surgical resection. Preoperative judgment is still a challenge. Previous studies have explored the preoperative diagnosis of MVI through the imaging features of HCC and specific tumor serum markers (Banerjee et al., 2015; Ryu et al., 2019), but there is still lack unified standard at present. Imaging examination such as computed tomography (CT), magnetic resonance imaging (MRI), positron emission tomography (PET) can visually observe the relationship between tumors and blood vessels, but it is often not sensitive enough to MVI. It is urgent to identify novel molecular markers for preoperative diagnosis of MVI.

Circulating tumor cells (CTCs) are tumor cells that shed from primary tumor focus and transvascular access to the circulatory system and finally colonized in metastatic site. CTCs have similar or even the same biological characteristics as primary tumors, which can be captured and pathological identified by blood separation methods (Yin et al., 2016; Bidard et al., 2019). Current research and speculation are insufficient to reasonably explain some clinical problems, especially the possibility that CTCs of HCC may form microvascular tumor thrombus from a single cell, and the possible leading role and molecular mechanism as a crucial component of tumor thrombus. Compared with other tumor markers, it is worth studying whether CTCs could accurately predict MVI in HCC. This review discusses the possible relevant mechanisms and application value of CTCs in predicting MVI of HCC.

## 2 Hepatocellular carcinoma microvascular invasion

MVI is a risk indicator related to HCC recurrence after hepatectomy or liver transplantation. The existence of MVI predict worse outcomes of HCC regardless of clinical treatment. Recent studies have proposed the importance of a preoperative assessment of MVI, which can be used to guide clinical therapy in patients with HCC.

### 2.1 Definition and incidence

With the innovation and progression of imaging diagnosis technology, surgery skills, targeting and the use of immune drugs, the overall survival time of HCC patients has been greatly improved, but the 5-year survival time is still far from satisfactory. One of the reasons is that vascular invasion has already occurred when the patients’ first diagnosis. Vascular invasion is categorized as either microscopic or macroscopic. HCC MVI is defined as clusters of cancer cells observed microscopically in vessels located in the tumor encapsulation and surrounding liver parenchyma (Roayaie et al., 2009). The incidence of MVI is between 15% and 57.1% (Rodríguez-Perálvarez et al., 2013). Even for small HCC (the maximum diameter of a single tumor nodule does not exceed 3 cm or the sum of the diameters of two tumor nodules does not exceed 3 cm), the incidence is as high as 18.1%–37% (Giuliante et al., 2012; Du et al., 2014). The appearance of MVI suggests more aggressive biological behavior of HCC and may be an important factor affecting the prognosis of patients.

### 2.2 Potential clinical value/clinical relevance of microvascular invasion

The vascular invasion of the tumor cells is a progressive process. If HCC is accompanied by macrovascular invasion, postoperative recurrence is probably inevitable. If only microvascular invasion occurs in patients with HCC, there is still great hope to achieve radical treatment.

MVI can usually be found in the tumor stroma, encapsulation and para-cancerous region. It is common in the portal vein branches and hepatic veins in the liver tissue adjacent to the cancer, the former is a potential source of intrahepatic metastases, while the latter is considered leading distant metastasis, including recurrence after liver transplantation (Erstad and Tanabe, 2019). Several retrospective studies have been revealed that the presence of MVI is a critical determinant of early HCC recurrence and prognosis (Lim et al., 2011; Lee et al., 2021). The risk and degree of MVI will also increase accompanied with the aggravation of disease. Therefore, MVI could be used as a predictor of tumor progression and prognosis.

Previous studies have found that when MVI is positive, the short-term recurrence rate of small HCC (≤2 cm) is higher (Yamashita et al., 2012), while for tumor diameter >2 cm, the long-term survival rate of patients is worse (Shindoh et al., 2013). Predicting MVI in patients with HCC before operation has a high clinical value for the choice of treatment. A multicenter clinical study on the surgical methods of early HCC with cirrhosis, anatomical hepatectomy could reduce the early recurrence of patients with pathological poor differentiation or with MVI, while there is no difference in the prognosis of patients with well differentiation or without MVI. Therefore, it is concluded that anatomic hepatectomy may be more beneficial to patients who are at high risk of MVI (Cucchetti et al., 2014).

MVI is an important factor indicating the prognosis of radical treatment for HCC, and has an unambiguous correlation with early recurrence and low survival rate (Lim et al., 2011; Zhang et al., 2018). Postoperative treatment combined with transcatheter arterial chemoembolization (TACE) can improve the prognosis of patients with HCC with MVI (Yang et al., 2021). TACE combined sorafenib treatment can improve the prognosis of HCC patients with MVI, while MVI negative patients cannot benefit from the above treatment methods (Peng et al., 2019). A decision-making model on whether 1024 patients with early HCC in the Eastern and Western countries could benefit from liver transplantation. The results showed that tumor diameter and number had a greater impact on the prognosis of liver resection, while MVI had a greater impact on the prognosis of liver transplantation. For those patients who were predicted high risk of MVI before operation, when both liver transplantation and hepatectomy are indicated, hepatectomy is the first choice (Vitale et al., 2014). In conclusion, preoperative prediction of MVI is helpful to guide the choice of clinical treatment strategies and the evaluation of prognosis in HCC.

### 2.3 Classification

According to the distribution and number of the microvascular invasion in tumor tissues, MVI could be classified to three degrees: M0, M1 and M2. M0 indicates no microvascular invasion is found in the section of tumor tissue, and the prognosis is generally favorable. M1 represents low-risk group, the number of microvascular invasions in all tissue sections was ≤5, and the microvascular invasion occurred in the liver tissue near the tumor. M3 represents high-risk group, the number of microvascular invasions in the tumor tissue section is more than 5, or the microvascular invasion occurs in the distal para-cancerous tissue (>1 cm) (Zhonghua Gan Zang Bing Za Zhi, 2015). When it is difficult to distinguish the satellite lesions and microvascular invasion in the adjacent liver tissue, they can also be included in the MVI classification.

### 2.4 Mechanism

Invasion and metastasis are the most essential biological characteristics of malignant tumors. Tumor metastasis is a complex evolutionary process driven by multi factors, involving tumor cells, microenvironment and the interaction between them (Ghajar, 2015). HCC is often caused by mutations accumulated in the genome of hepatocytes under the stimulation of various factors in microenvironment such as chronic inflammation and environmental toxins. Cells in the tumor microenvironment produce a variety of cytokines, which could stimulate the proliferation and transformation of HCC cells, induce the formation of vascular endothelial cells, and recruit immune cells. Meanwhile, these cells can increase the secretion of cytokines and accelerate the occurrence, development, invasion and metastasis of tumors (Ye et al., 2014). Besides, tumor blood vessels could provide necessary nutrition supply and immune microenvironment for tumor growth, and accelerate the malignant transformation of tumor (Rodríguez-Perálvarez et al., 2013).

Epithelial-mesenchymal transition (EMT) refers to the transformation process of cells from epithelial phenotype to mesenchymal phenotype, deprivation of cell polarity and the ability to adhere to the basement membrane, and acquirement malignant phenotypes such as proliferation, migration, invasion, and apoptosis resistance (Kong et al., 2022). Recent studies have discussed the relationship between EMT and MVI in HCC. EMT related transcription factors, protein markers (such as E-cadherin, N-cadherin and Vimentin), epigenetic abnormalities (such as noncoding RNAs) could be correlated with vascular invasion, intra/extrahepatic metastases, and early recurrence in HCC patients (Zhou et al., 2012; Mima et al., 2013; Cancer Genome Atlas Research Network, 2017; Wan et al., 2017).

In short, MVI is a process in which HCC cells continuously stimulated by inflammation and other factors and activated by several signaling pathways such as PI3K/AKT, Wnt/β-catenin (Whittaker et al., 2010). And then these tumor cells with invasive phenotype gradually destroy the surrounding tissue structure by degradation of the basal membrane (Wu et al., 2011). At last, tumor cells infiltrate the surrounding stroma, vascular wall and invade the microvascular lumen to form microvascular thrombus by resistance to survival pressure such as blood shear stress, anoikis, oxidative stress and immune surveillance (Hanahan and Weinberg, 2011). The tumor thrombus could further proliferate to form macroscopic thrombus and then shed and spread into the circulation system to form distant metastasis. In view of the important clinical significance of MVI in HCC, it is necessary to accurately identify and predict MVI before operation. However, unlike macrovascular invasion, traditional imaging examinations are hard to precisely evaluate MVI, and histopathological examination is still the only reliable method to evaluate MVI. Therefore, it is urgent to develop novel reliable factor for preoperative diagnosis and evaluation of MVI.

## 3 Circulating tumor cells

In the 1830s, “seed and soil” theory of tumor metastasis had been put forward by Steven Paget, which guide the clinical treatment till now (Paget, 1989). In recent years, CTCs that escape from the primary tumor, intravasation into and survival in the blood circulation, extravasation the circulation and colonization growth in the metastatic site are considered to be a vital indicator for postoperative recurrence and metastasis of malignant tumors and show extremely significant clinical value in tumor diagnosis, dynamic detection of treatment response and prognosis evaluation (Yu et al., 2013; Lin et al., 2021). From the recent research on the correlation between risk factors and CTCs, CTCs are not associated with obesity and may be independent prognostic indicators for PFS in patients with HBV-related HCC (Ye et al., 2018; Tzschaschel et al., 2023). CTCs can be directly isolated from patients’ peripheral blood without invasive tumor biopsy, and can dynamically monitor patients’ prognosis (Plaks et al., 2013). The molecular map (mutations in the epidermal growth factor receptor gene (EGFR), phosphatidylinositol 3-kinase gene (PIK3CA), estrogen receptor gene (ESR1), fibroblast growth factor receptor gene (FGFR2), etc.) obtained from the isolated CTCs is closely related to therapeutic effect (Maheswaran et al., 2008), and may reveal candidate target genes for drug therapy (Yu et al., 2014). Although development in technology have enabled people to exploit analytical methods evaluating disease progression and treatment response by using CTCs, there are still many unsolved aspects about the changes of biodynamics, the number and molecular phenotype of CTCs over time (Leong et al., 2015). Due to these limitations, there is no recommendation of CTCs to guide clinical treatment due to the lack of clinical practical data based on CTCs trials (Harris et al., 2016; Sepulveda et al., 2017).

### 3.1 CTCs and microvascular invasion

CTCs are associated with the occurrence of macrovascular invasion and the prognosis of patients (Li et al., 2014). However, available study between CTCs and microvascular invasion is still very limited. Zhou et al. suggested that patients with preoperative CTC counts ≥5 were more likely to have MVI (Zhou et al., 2020). According to the previous studies, we speculate that cancer cells actively disseminated into the bloodstream from the primary tumor site or passively released into the circulation system due to surgical manipulation may be the core factors of MVI.

### 3.2 Hepatocellular carcinoma CTCs initiate coagulation in peripheral blood to form initial circulating tumor microembolus

**FIGURE 1:**
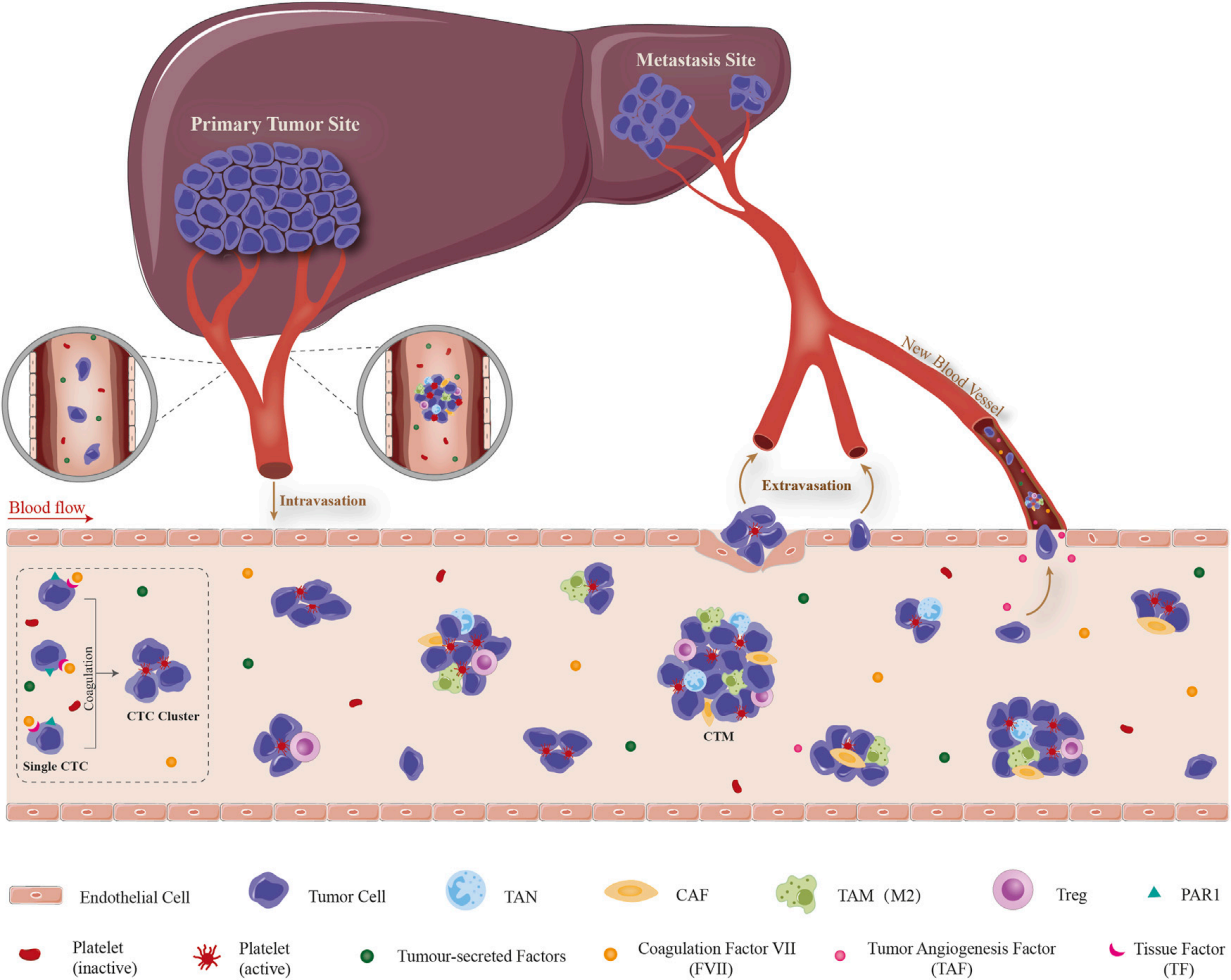
Schematic illustration of dissemination, survival and extravasation of CTC/CTM from the circulation which consequently promoting the formation of MVI. The complex process includes the detachment of tumor cell from the primary site, intravasation/survival in the circulation as CTCs and interaction with cellular and non-cellular components dissociated in blood circulation, extravasation from the circulation, attachment to and targeted colonization of the metastatic site. Detached CTC/CTM from HCC tissue circulate in peripheral circulation system. TF which highly expressed on the surface of CTC promotes contact and binding with the coagulation factors in the blood and triggers the coagulation cascade reaction. This local coagulation reaction could ultimately promote the mutual aggregation and adhesion of HCC CTC, and finally form CTM. Moreover, tumor-derived cytokines in the circulatory system participate in the recruitment of CAF, TAM, Treg, etc., and the formation of a special microenvironment which suitable for the survival/growth of HCC CTC/CTM, resistance to anoikis, promotion of neovascularization and immune evasion. It provides the possibility for CTC to grow into MVI, transendothelial migration and home in liver. CTC: circulating tumor cell, CTM: circulating tumor microembolus, TF: tissue factor, CAF: cancer-associated fibroblast, TAM: tumor-associated macrophage, TAN: tumor-associated neutrophil, Treg: regulatory T cell, PAR1: protease-activated receptor 1.

In the circulation system, CTCs mostly exist in the form of single cells or multiple tumor cells. Single CTCs have lost original epithelial properties and acquired invasive phenotype via EMT process (Thiery et al., 2009). Tumor cells which composed of two or more (even more than 50) CTCs, have retained some of original epithelial phenotype, called CTCs clusters or CTM (Cheung et al., 2016; Dongre and Weinberg, 2019). Other types of cells such as fibroblasts, leukocytes, endothelial cells, parietal cells and platelets could also participate in the formation of CTM, which playing a role similar to the microenvironment (Krebs et al., 2014; Castro-Giner and Aceto, 2020). To confront with a series of environmental stress including shear forces, anoikis, oxidative stress and immune surveillance, only a very small number of CTCs could survive, reach the colonization site and form metastases finally (Hanahan and Weinberg, 2011). Previous studies have shown that cancer cells in CTM are easy to survive in bloodstream, have a higher potential of distant metastasis than single CTC, and are also a crucial indicator in evaluating poor prognosis and chemotherapy resistance. There are two possible mechanisms for the formation of CTM: one is that a single tumor cell shed from the primary site invades the stroma, spread into the blood vessels through the perivascular space, and aggregates into clusters to form CTM (Cheung et al., 2016; Castro-Giner and Aceto, 2020). Second, tumor cells form clusters directly in the primary focus and invade microvascular after shedding (Aceto et al., 2014; Cheung et al., 2016). As far as HCC is concerned, recent clinical studies including our group showed that high content of CTCs is closely related to MVI (Xu et al., 2011; Li et al., 2013). Whether it is the HCC CTCs that cause the formation of vascular tumor thrombi or the dissemination of HCC cells from vascular tumor thrombi that leads to the increase of circulating CTCs? We speculate that both situations may exist at the same time. Therefore, a scientific question arises: does HCC CTCs actively trigger adhesion and coagulation reactions, stay in microvascular and/or portal vein with blood flow after the formation of CTM, and then form MVI or even PVTT? The complex process includes the detachment of tumor cell from the primary site, intravasation/survival in the circulation as CTCs and interaction with cellular and non-cellular components dissociated in blood circulation, extravasation from the circulation, attachment to and targeted colonization of the metastatic site was detailed illustrated in Figure 1.

#### 3.2.1 Tissue factor

Emerging studies have supported that the activation of coagulation involved with malignancy and tumor progression (Hu et al., 2004; Gil Bernabé et al., 2013). TF is the only type I transmembrane glycoprotein expressed on the cell membrane surface of coagulation factors. As the cell surface receptor of coagulation factor VII (FVII), TF could bind and activate FVII, form TF/FVII complex, and then trigger the downstream coagulation cascade leading to thrombin generation and clot formation. It has been demonstrated that TF play an important role in hemostatic system and cancer progression (Ruf, 2012; Gil‐Bernabé et al., 2013). Zhou et al. reported that TF is over-expressed in both plasma and tissues in HCC patients, and it is closely related with lymphatic metastasis, extrahepatic metastasis and portal tumor thrombus (Zhou et al., 2011). TF expression is upregulated in more aggressive EMT^+^ cells and CTCs, providing tumor cells with enhanced coagulant properties that facilitate early steps of metastatic colonization (Bourcy et al., 2016). Considering its angiogenic role in tumors (Nakasaki et al., 2002; Fernandez et al., 2004), it is probably involved in HCC metastasis, however the underlying mechanism is not yet completely known. We speculate that CTCs released from HCC also express TF on the surface of cell membrane. TF exposed to blood could activate FVII, initiate exogenous coagulation pathway and finally form CTM. HCC CTCs have procoagulant activity, and the high expression of TF in HCC CTCs may mediate the formation of CTM in circulation.

#### 3.2.2 Protease activated receptors

During the progression of vascular tumor thrombus after the formation of CTM by HCC CTCs, what is the mechanism by which HCC CTCs acquire the proliferative potential and grow into microscopic or macroscopic vascular tumor thrombus? Studies have shown that TF could regulate the proliferation and growth of HCC cells by activating PARs, which become a promoter of the formation and development of vascular tumor thrombi. TF-FVIIa complex on the cell membrane could mediate signal transduction by activating PARs. FVa and thrombin, the products of coagulation cascade, could also recognize one or more PARs receptors which belonging to the superfamily of G protein coupled receptors, and further amplifying PARs mediated signal transduction. Activation of these signal transduction can produce angiogenic factors, cytokines and adhesion factors, which promote tumor cell proliferation, apoptosis resistance, adhesion, invasion and tumor angiogenesis (Vizurraga et al., 2020), even rouse dormant tumor cells by altering the genome (Dragoni et al., 2020). Studies have shown that PARs are highly expressed in tumor tissues and is positively correlated with the degree of malignancy, invasion and metastasis potential (Vizurraga et al., 2020). We believe that after initiating the formation of CTM, TF also promotes the proliferation and growth of HCC cells through PARs signaling pathway, thus becoming a crucial promoter for remodeling the microenvironment and promoting the formation and progress of MVI. Mouse models and experimental therapeutics have demonstrated crucial roles for TF initiated cell signaling in the pathogenesis of cancer. On tumor cells, the TF-VIIa binary complex regulates activation of protease activated receptor (PAR) and thereby shapes the tumor microenvironment by inducing a series of proangiogenic (such as VEGF, Cyr61, VEGF-C, CTGF) and immune modulating cytokines (such as GM-CSF, M-CSF), chemokines (such as CXCL1, IL-8), and growth factors (such as EGF) (Schaffner and Ruf, 2009; Wojtukiewicz et al., 2015).

##### 3.2.3 Platelet 

Platelets are small pieces of cytoplasm that released from the cytoplasm of mature megakaryocytes in bone marrow which have the function of hemostasis and promoting wound healing. The life span of platelets is only 8–10 days and the normal platelet count is between 150,000 and 450,000 platelets per microliter of blood (Závodszky et al., 2019). Studies have shown that platelets are participants in multi-step of tumorigenesis, including tumor growth, extravasation and metastasis (Jurasz et al., 2004; Erpenbeck and Schön, 2010). Mechanisms by which platelets assist hematogenous metastasis depend on the ability of platelets to act in protecting tumor cells within the blood stream.

##### 3.2.3.1 Platelets assist tumor cells in dissemination

The prerequisite for tumor metastasis is that tumor cells can survive in circulation. After tumor cells enter the peripheral blood, a large number of CTCs will be rapidly destroyed. Only a small number of CTCs escape the clearance of microenvironment. Platelets are closely related to the process of tumor metastasis. Previous studies revealed that tumor cells could secrete ADP, thrombin, matrix metalloproteinases (MMPs), interleukin-6 (IL-6), TF and other components to activate platelets (Ward et al., 2018; Zarà et al., 2018). Activated platelets play a crucial role in the dissemination and metastasis of tumor cells and the thrombosis of patients (Riedl et al., 2017). On one hand, activated platelets could enhance the adhesion of tumor cells and weaken the shear force from the interaction between blood and blood vessels, as well as the immune recognition and killing effect of immune cells (such as NK cells). On the other hand, platelets can also secrete a series of cytokines and other regulatory factors such as adenosine diphosphate (ADP), integrin α6β1, promote the survival, hematogenous metastasis of tumor cells and the colonization (Haemmerle et al., 2017).

##### 3.2.3.2 Platelets provide nutritional support for tumor progression

In 1971, Folkman put forward the theory that “tumor growth and metastasis depend on neovascularization” (Sherwood et al., 1971). Neovascularization is a common event in tumor progression which could provide essential nutrients and oxygen for further lesions, and facilitate the dissemination and metastasis in distant site of tumor cells in circulation (Folkman, 2002). Tumor angiogenesis is a complicated network, which requires tumor cells and other cells in tumor microenvironment, as well as the recruitment of cells from bone marrow. Platelets may contribute to angiogenesis during tumor development. Platelets are the main transport carrier of pro-angiogenic (vascular endothelial growth factor (VEGF), platelet-derived growth factor (PDGF), transforming growth factor beta (TGF-β), basic fibroblast growth factor (bFGF), MMPs) and anti-angiogenic factors (angiopoietin-1 (ANGPT1), sphingosine1-phosphate (S1P), serotonin, thrombospondin-1 (THBS1)) (Coppinger et al., 2004; Smyth et al., 2009; Gay and Felding-Habermann, 2011; Yan and Jurasz, 2016), which regulate the angiogenic process. VEGF is one of the most important angiogenic proteins, which is transported and released by platelets. VEGF could directly loosen the junction between adjacent endothelial cells and thereby increasing its permeability (Saharinen et al., 2011), meanwhile stimulate the angiogenesis of distant metastases (Dymicka-Piekarska et al., 2021). In short, platelets play an important role in tumor angiogenesis which is a key step of tumor growth and metastasis.

##### 3.2.3.3 Platelets help tumor cells escape the attack of the immune system

The cells of the immune system involved in tumor immune surveillance mainly include natural killer cells (NK cells), cytotoxic T cells (CTL), tumor associated macrophages (TAMs) and dendritic cells (DC) which related to tumor inhibition. Therefore, immune escape is a key step for tumor cells to achieve metastasis. The anti-tumor effect of NK cells is largely affected by platelets (Clar et al., 2019). In the circulation, activated platelets could aggregate around tumor cells, inhibit the function of NK cells, and facilitate immune escape of tumor cells.

Platelets could induce the release of soluble NKG2D ligands in tumor cells, thus avoiding the recognition of tumor cells by NK cells, meanwhile inhibiting degranulation and secretion of inflammatory factors such as IFN-γ of NK cells (Cluxton et al., 2019). This mechanism effectively inhibits the immune clearance of tumor cells by NK cells. Besides, platelet-derived TGF-β inhibited the function of NK cells through CD226/CD96-CD112/CD155 axis (Cluxton et al., 2019). Platelets can strongly inhibit the expression of CD226 and CD96 on the surface of NK cells and their related ligands, thus further enhancing the inhibitory effect of platelets on NK cells (Clar et al., 2019). These studies have confirmed that CTCs could be protected by platelets and hided from NK cells and immune surveillance, and provided the possibility for subsequent metastasis and colonization in distant sites.

##### 3.2.3.4 Platelets assist tumor cells in metastasis and colonization

Studies have shown that the surrounding of platelets to the CTCs enhances the adhesion of the CTCs to the vessel wall by reducing the exposure time of the CTCs to the shear stress and immune assault which are critical factors for the viability of CTCs (Anvari et al., 2021). The activation of platelet endothelial cell adhesion molecule-1 (PECAM-1) can regulate the junction between adjacent endothelial cells, which is essential to adhesion, transendothelial migration and extravasation of CTCs (Leblanc and Peyruchaud, 2016; Wojtukiewicz et al., 2017). VEGF could be secreted by CTCs to expand the space between adjacent endothelial cells which results in the permeabilization of microvessels (Stoletov et al., 2007). The interaction between CTCs and platelets greatly increases the possibility of microvascular extravasation (Lou et al., 2015). Moreover, it has been demonstrated that platelets regulated tumor vascular integrity, as their depletion selectively rendered tumor vessels highly permeable and caused massive intra-tumoral hemorrhage, eventually affected tumor progression (Volz et al., 2019). In conclusion, platelets not only directly surround CTCs to protect them from clearance by flow shear stress and immune cells in the bloodstream, participate in tumor neovascularization by transporting cytokines such as VEGF and provide sufficient nutrients for tumor progression, but also facilitate adhesion to the endothelium and transendothelial migration of CTCs by regulating tumor vascular integrity, eventually leading to metastasis and colonization. Although functions of platelets in tumor metastasis have been revealed in recent years, the specific mechanisms that initiate and maintain the interaction between platelets and CTCs are still need further investigation.

To sum up, the prerequisite for tumor cell metastasis is that the tumor cells can survive in circulation. After tumor cells enter the peripheral blood, a large number of CTCs are rapidly destroyed and cleared. A small number of CTCs interact with platelets to establish a partial physical barrier of immune attack, interfere with the recognition of tumor cells by natural killer cells, assist in tumor nutrition supply, eventually promote tumor cell survival and metastasis.

#### 3.2.4 Cancer associated fibroblasts

Tumor cells initiating metastasis requires cross-talk with stromal cells, especially with CAFs, which is known as the most abundant cell types within the microenvironment (Majidpoor and Mortezaee, 2021). CAFs are involved in tumor progression and invasion by secreting MMPs which contribute to Extracellular matrix (ECM) degradation (Kalluri and Zeisberg, 2006). Furthermore, CAFs could induce EMT in cancer cells and promote tumor angiogenesis through secretion of cytokines such as TGF-β and VEGF (Liao et al., 2009). Besides, cytokines derived from CAFs provide a survival advantage to tumor cells and assist them in escaping the clearance of immune system (Liao et al., 2009). Based on the latest research, CAFs-derived cytokines such as HGF, IL-6, CCL2, CXCL1, CXCL8, SCGF-b, and VEGF were more likely to induce and maintain stem properties of HCC cells (Lau et al., 2016; Li et al., 2019).

In the circulation, CTC clusters can contain a heterogeneous group of cells including CAF, immune cells, epithelial cells, and platelets (Ortiz-Otero et al., 2021). Recent studies have also indicated that abundant circulating CAFs could be observed in the bloodstream, CTCs carrying CAFs as their own “soil” during circulation, thus evading cell death and facilitating the establishment of a metastatic niche at distant sites (Duda et al., 2010; Ao et al., 2015). In brief, we can speculate that CAFs can affect CTCs from following two aspects. On one hand, promoting the survival of CTCs by secreting a series of cytokines, inducing and maintaining stem properties of HCC CTCs. On the other hand, adhesion with CTCs directly and providing a microenvironment for resistance to the survival stress and immune clearance in the circulation, meanwhile promoting tumor neovascularization and facilitating the final colonization and metastasis of CTCs.

### 3.3 Hepatocellular carcinoma CTCs interact with immune cells in circulation to escape immune surveillance

**FIGURE 2:**
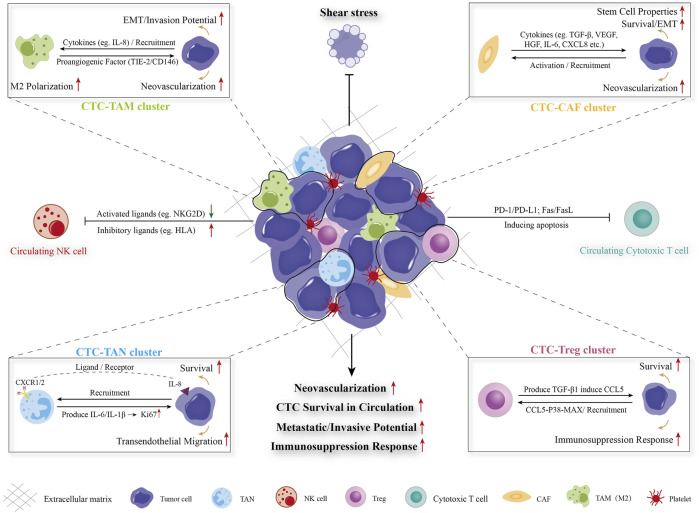
Diagram of CTC-blood interactions during haematogenous dissemination. CTC/CTM spreads though the circulation which constantly interacts with stromal cells (such as CAF and extracellular matrix) and a variety of cancer-associated immune cells (TAM, TAN, NK, Treg, etc.), which could enhance the resistance of survival pressure including fluid sheer stress, anoikis, immune clearance, etc., and facilitate the metastatic/invasive potential, neovascularization and immune escape ability of CTC/CTM. CTC: circulating tumor cell, CTM: circulating tumor microembolus, CAF: cancer-associated fibroblast, TAM: tumor-associated macrophage, TAN: tumor-associated neutrophil, Treg: regulatory T cell, NK cells: natural killer cells, EMT: epithelial-mesenchymal transition.

Mount studies have considered CTCs as the “seeds” for intrahepatic and extrahepatic metastasis in HCC. During dissemination process, CTCs are exposed to multiple types of survival stress in circulating microenvironments, including shear forces, anoikis, oxygen/nutrient insufficiency, and immune surveillance. Among them, the successful evasion of immune cells mediated killing is crucial for CTCs survival and dissemination. The specific process and mechanisms of CTC/CTM spreads though the circulation which constantly interacts with stromal cells (such as CAF and extracellular matrix) and a variety of cancer-associated immune cells (TAM, TAN, NK, Treg, etc.), which could enhance the resistance of survival pressure including fluid sheer stress, anoikis, immune clearance, etc., and facilitate the metastatic/invasive potential, neovascularization and immune escape ability of CTC/CTM were detailed illustrated in Figure 2. The important mechanisms of CTC in interacting with different immune cells in forming MVI have been summarized in Table 1.

**TABLE 1:** Types of immune cells involved in the formation of MVI with HCC CTC.

Types of immune cells	Mechanisms	References
TAMs	M2 polarization and accumulation of TAMs stimulated by HCC-derived IL-8, EMT↑, invasive potential **↑**	Ao et al. (2015)
	Direct adhesion with and protect CTCs from survival stress such as shear stress, immune clearance and anoikis	Li et al. (2019); Duda et al. (2010)
	Neovascularization**↑** by expressing proangiogenic factors (TIE-2/CD146)	Duda et al. (2010)
TANs	Direct adhesion with CTCs and produce IL-6 and IL-1B, Ki67**↑**and the survival rate of CTC clusters in circulation ↑	Murphy (1997); Huh et al. (2010)
	Expressing CXCL5/CXCR2 axis, EMT↑, invasive potential↑	Kim et al. (2009)
	Releasing cytokines such as (Oncostatin M), angiogenesis and metastasis potential ↑	Liu et al. (2019)
NK cells	Activated ligands (e.g., NKG2D) **↓** and inhibitory ligands (e.g., HLA) **↑**, cell-cell adhesion genes↑, survival and metastasis of CTCs**↑**	Xiao et al. (2018)
	Participating in immunosuppression and neovascularization, survival and metastasis of CTCs**↑**	Lanier (2008); Lo et al. (2020)
Cytotoxic T cells	Inducing apoptosis by PD-1/PD-L1 or Fas/FasL (expressed on the surface of CTCs)	Deepak and Acharya (2010); Mego et al. (2016); Ye et al. (2017); Liu et al. (2021)
Tregs	Producing TGF-β1, inducing CCL5 expression in CTCs; recruiting Tregs via TGF-β1/CCL5/p38/MAX signaling, survival of CTCs**↑**	Gruber et al. (2013)

Abbreviations: MVI, microvascular invasion; HCC, hepatocellular carcinoma; CTC, circulating tumor cell; TAMs, tumor-associated macrophages; IL-8, interleukin 8; EMT, epithelial-mesenchymal transition; TANs, tumor-associated neutrophils; IL-1β, interleukin-1β; CXCL5, CXC-chemokine ligand 5; CXCR2, CXC-chemokine receptor 2; NK, cells, natural killer cells; PD-1, programmed death-1; PD-L1, programmed death-ligand 1; Fas, recombinant factor related apoptosis; FasL, recombinant factor related apoptosis ligand; Tregs, regulatory T cells; TGF-β1, transforming growth factor-β1; CCL5, C-C motif chemokine ligand 5.

#### 3.3.1 Tumor-associated macrophages

Macrophages in the tumor microenvironment (TME), usually termed to as TAMs, are one of the most abundant types of cells, and exhibit multiple phenotypes and functions in response to variable biological signals derived from tumor and stromal cells (Lin et al., 2019). It has been reported that TAMs serve as a crucial component of TME with immune-protective, pro-angiogenic and invasiveness-supporting actions in human cancers (Qian and Pollard, 2010). Recent studies have revealed that TAMs are involved in the dissemination, proliferation, and neovascularization of metastases of CTCs. Xiao et al. found that HCC-derived IL-8 attracted more TAMs to the local microenvironment, displaying enhanced cytokine secretion and phagocytosis. IL-8 stimulated the M2 polarization of TAMs, which promoted the EMT and invasive potential of HCC cells (Xiao et al., 2018). It provided a clue that TAMs may be involved in the initiation of tumor metastasis and blood dissemination of CTCs. Adams DL and colleagues reported that TAMs originating at the tumor site disseminate into the circulation in large numbers and bound to CTCs and disseminate together in circulation which suggested that TAMs presented some participatory function in the migration of CTCs in the bloodstream of advanced-stage patients. Meanwhile, proangiogenic factors (TIE-2/CD146) could be detected on TAMs indicating that TAMs have the ability to neovascularize a metastatic microenvironment (Adams et al., 2014). It is suggested that TAMs serve as prominent metastasis promoters in the TME. On the one hand, TAMs could protect CTCs from the destruction of survival stress such as shear stress, immune clearance and anoikis by direct adhesion in the circulation. On the other hand, TAMs may express or secrete angiogenesis related factors, thereby facilitating tumor metastasis.

#### 3.3.2 NK cells

CTCs interact with immune cells and metastatic microenvironments, survival signaling, and chemotherapeutic resistance. Among immune cells, NK cells can, directly and indirectly, interact with CTCs to control cancer metastasis.

NK cells, a vital member of the innate immune system, which can rapidly recognize and destroy tumor cells or virus-infected cells without previous sensitization or priming. The killing activity of NK cells is regulated by the interplay between the inhibitory receptors and activating receptors (Lanier, 2008; Lo et al., 2020). Studies have shown that the decrease in the number and impaired activity of NK cells are significantly related to tumor metastasis in mouse models and in patients (Gras Navarro et al., 2015; López-Soto et al., 2017). The latest research has suggested that CTC clusters increased expression of NK cell inhibitory ligands and decreased expression of NK cell activating ligands, accompanied by an upregulation of cell-cell adhesion genes which eventually leading to the survival and metastasis of CTCs (Lanier, 2008). Moreover, NK cells in the peripheral circulation also participate in immunosuppression and neovascularization in solid tumors, thereby promoting tumor metastasis (Bosi et al., 2018; Bruno et al., 2018). NK cells could be classically divided into IFN-γ-producing CD56^low^CD16^high^ and metastasis-promoting CD56^high^CD16^low^ subsets with different functions in tumor immunity (Dianat-Moghadam et al., 2018). While CD56^low^ NK cells have strong killing effect on tumor cells, and CD56^high^ NK cells are presented with immunosuppressive features and negatively correlated with overall survival that are found in metastatic disease and can drive angiogenesis (Bosi et al., 2018; de Jonge et al., 2019).

#### 3.3.3 Tumor-associated neutrophils

A better understanding of the interaction between tumor cells and immune cells is essential for the development of tumor immunotherapy. Neutrophils are the most abundant circulating leukocyte and the first responders to sites of acute tissue damage and infection. In chronic inflammation, neutrophils could persist in tissues and are closely related to tumor progression (Powell and Huttenlocher, 2016). However, the role of neutrophils in the tumor microenvironment remains controversial.

Neutrophils express the chemokine receptors (CXCR1 and CXCR2) which are crucial for chemotaxis (Murphy, 1997). Cancer cells express various ligands for these receptors that facilitate recruitment of neutrophils. As previous studies mentioned, CTC-derived IL-s accelerate the “tumor self-seeding” process by attracting neutrophils. The recruited neutrophils interact with CTCs and help CTCs adhering to the vessel wall, promoting transendothelial migration, and producing MMPs to remodel ECM, eventually facilitating CTCs colonization and metastases establishment (Kim et al., 2009; Huh et al., 2010; Liu et al., 2019). TANs could produce IL-6 and IL-1B which upregulate the expression of Ki67 (a proliferative marker) and promote the survival rate of CTC clusters in circulation (Kumagai et al., 2020). The CXCL5/CXCR2 axis was also found to be involved in EMT process of HCC cells and contributes to tumor invasion (Zhou et al., 2015). Besides, neutrophils also contribute to angiogenesis and metastasis by releasing cytokines such as Oncostatin M which induces VEGF production (Queen et al., 2005). An *In vivo* model revealed that neutrophils directly interact with CTCs to support the CTC cluster formation and cell cycle progression in circulation and to accelerate metastasis seeding (Szczerba et al., 2019). Overall, TANs escort the formation and survival of CTCs/CTC clusters until they reach to the target organ and establish colonization for metastasis.

#### 3.3.4 T lymphocyte (T cells)

Immune disorders often occur in tumors, leading to uncontrolled growth of tumor cells. One of the most important reasons is attributed to the functional damage of T cells *in vivo*. T cells, which derived from pluripotent stem cells of bone marrow, is commonly divided into CD4 and CD8 subtypes according to its surface markers and functions (Gutcher and Becher, 2007). T cells can be recycled through lymphatic vessels, peripheral blood and tissue fluid to perform the functions of cellular immunity and immunoregulation. The classification of CD4^+^ T cells, which also called Helper T cells (Th), includes Th1, Th2, Tfh, Th17, and Treg with specific transcription factors and different secreting cytokines (Sekiya et al., 2021; Xydia et al., 2021). CD4^+^ T cells expressing cell surface CD4, which could be activated by the peptide antigen presented by major histocompatibility complex class Ⅱ (MHC Ⅱ). CD8^+^ T cells, also called cytotoxic T cells expressing cell surface CD8 are the most powerful effectors in the anti-tumor immune response (Raskov et al., 2021). CD8^+^ T cells recognize tumor antigens presented by MHC I and effectively destroy surrounding tumor cells after activation (Deepak and Acharya, 2010).

In recent years, with the continuous breakthrough of tumor immunotherapy, the correlation between CTCs and T cells has been paid more and more attention. Liu reported that there was a significant negative correlation between the proportion of CD8^+^ T cells and CTCs count after radiotherapy in non-small cell lung cancer (Liu et al., 2021). The high CTC levels were found to be correlated with a reduction in adoptive CD3^+^/CD4^+^/CD8^+^ T cells in breast and lung cancer patients and positively correlates with metastasis (Mego et al., 2016; Ye et al., 2017). High expression of programmed death-ligand 1 (PD-L1) in tumor cells inhibit the killing effect of immune system by inducing apoptosis of cytotoxic T cells and increasing regulatory T cells (Ostroumov et al., 2018; Jiang et al., 2019). Chalfin reported that PD-L1 expressed on CTCs and the presence of PD-L1^+^ CTC subtype, low CD4 and CD8 T-cell counts had shorter survival in metastatic genitourinary cancer patients. CTCs reduce cytotoxic T lymphocyte immune responses through downregulating MHC I expression on CD8^+^ T cells, and PD-L1 which expressed on CTCs that binds to PD-1 in CD8^+^ T-cell leading to the inhibition of their antitumor activity (Dianat-Moghadam et al., 2020). In addition, FasL expressed on the surface of CTCs can bind with Fas receptor on the surface of cytotoxic CD8 T^+^ or CD4^+^ cells to activate the extrinsic apoptosis and T cell exhaustion (Gruber et al., 2013).

#### 3.3.5 Regulatory T cells (Tregs)

During dissemination and metastasis in the circulation, CTCs need to escape the surveillance and elimination by the immune system. The mechanism of immune evasion is usually consisted of two ways: of tumor cells occurs in two ways: hiding the self-antigens information that can be recognized by the immune system; inhibiting immune effector cells or inducing suppressive immune cells by the secretion of immunosuppressive cytokines (Bhatia and Kumar, 2014). Tregs belong to CD4 T cell subgroup which participate in the immune escape of tumor cells by the induction of immunosuppression (Shevach, 2018). Tregs have the ability to infiltrate and accumulate in tumor tissues which are closely related to poor prognosis of patients (Shang et al., 2015; Sun et al., 2021). Do Tregs exist in the circulatory system, and whether they have an impact on the survival and metastasis of CTCs? The latest research has revealed that CCL5 (known as an immunosuppressive chemokine) is overexpressed in CTCs which form an immunosuppressive microenvironment by recruiting Tregs in the circulation via TGF-β1-p38-MAX signaling, eventually promoting CTC survival and metastatic seeding (Sun et al., 2021). CTC-related lymphocytopenia in tumor patients is correlated with increased numbers of Tregs (Mego et al., 2016). Therefore, as an important type of immune cells, Tregs exert tumor immunosuppressive responses which could be recruited into circulation, protecting CTCs from elimination by effector T cells and escorting CTCs to the metastatic site for colonization.

### 3.4 CTCs which form MVI ultimately breaking through the vascular barrier and lead to metastasis through transendothelial migration

CTCs which survival from attacks of mechanical stress and immune cells in the circulation, extravasate the vascular endothelium is also an essential step of metastasis. Unfortunately, specific steps and regulating mechanisms of CTCs extravasation are not yet well understood. Accumulating studies have suggested that CTCs extravasation is a complicated multi-step process which involves adhesion to the endothelium, modulation and disintegration of endothelial barrier, and transendothelial migration to adjacent tissues (Strilic and Offermanns, 2017).

Adhesion process between CTCs and the endothelium is essential and should be the initial step of extravasation and metastasis which involves the cooperative participation of multiple signaling pathways including selectins, cadherins, integrins, immunoglobulin superfamily receptors, chemokine and its receptors, growth factors, and mechanical factors (Reymond et al., 2013; Strilic and Offermanns, 2017).

After adhesion to the endothelium, the endothelial cell junctions and vascular permeability could be modulated by CTCs and other components in tumor microenvironment through either direct contact or secreted cytokines and extracellular substance (such as growth factors, chemokines, and exosomes) (García-Román and Zentella-Dehesa, 2013; Xu et al., 2018). A single CTC can squeeze through the endothelial cell barrier by the way of diapedesis; CTC clusters can remodel vascular endothelial cells through angiopelosis, forming pocket-like structures around CTC clusters to facilitate their extravasation out of the blood vessels (Allen et al., 2019; Cheng and Cheng, 2021). In the latest research, several novel molecules have been identified which promote the extravasation of CTCs in HCC. Zhang and colleagues reported that Rho family guanine nucleotide exchange factor 37 (ARHGEF37) enhanced the extravasation and lung metastatic capability of HCC cells through promoting the formation of invadopodia, consequently resulting in disruption the interaction between endothelial cells and pericytes (Zhang et al., 2022). Peng et al. found that chloride intracellular channel 1 (CLIC1) recruited PIP5Ks to the leading edge of plasma membrane, accompanied with the activation of talin and integrin α4β1 and α6β4 to initiate the assembly of nascent cell-matrix adhesions and adhesion-mediated signaling for actin cytoskeleton remodeling to form lamellipodia and invadopodia, and eventually lead to extravasation and metastasis of circulating HCC cells (Peng et al., 2021).

## 4 Conclusion and future perspectives

As one of the high recurrent malignant tumors, HCC develops rapidly with high mortality rate. Postoperative prognosis is frustrating mainly due to early recurrence, which significantly restricts the long-term survival of patients. At present, there is a lack of effective postoperative therapeutic strategies to prevent recurrence. Therefore, precise preoperative decision-making for HCC patients is extremely important. It has been confirmed that there are many indicators that influencing postoperative recurrence, such as hepatitis B virus, tumor diameter, tumor number and PVTT. Meanwhile, MVI as a risk factor of postoperative recurrence cannot be ignored. Precisely predicting MVI before operation has a certain guiding significance for the choice of surgical methods.

The vascular invasion of HCC may originate from the direct invasion of portal vein or small branches of hepatic vein, which adjacent to or not adjacent to the main tumor, or even appear in the portal vein or hepatic vein of the contralateral liver lobe (Matsumoto et al., 2009; Sumie et al., 2014b; Liu et al., 2018; Zhang et al., 2018; Sheng et al., 2020). Regardless of the relationship between the site of occurrence and the main tumor, vascular invasion may be caused by the adhesion of CTCs shed into the blood, and the continuous proliferation in the microvascular or portal vein to form cancer cell nests or tumor thrombi (Matsumoto et al., 2009; Sumie et al., 2014b; Zhang et al., 2018). Up to now, the definite mechanism of MVI in HCC are not fully elucidated. It is widely recognized that the formation of MVI is a complex process with multi-step regulation, such as pathological anatomy of liver cirrhosis, hemodynamics and tumor molecular biology (Liu et al., 2018; Zhang et al., 2018). Current studies are mainly based on the pathological anatomy, and retrospective analysis the correlation between vascular invasion and recurrence, metastasis and prognosis (Matsumoto et al., 2009; Shen et al., 2011; Lei et al., 2016; Liu et al., 2018; Krishnan et al., 2021). However, these studies or hypotheses fail to reasonably explain many clinical problems. For example, not all vascular invasion and the main tumor have a dependent anatomical relationship; vascular invasion could also occur in the liver where hemodynamics has not changed significantly. Due to the limitations of experimental methods and models, few studies have carefully verified the relevant hypotheses.

In recent years, CTCs have been considered to be closely related to the recurrence and metastasis of HCC. Different from the traditional biopsy, CTCs can be used as a novel repeatable and non-invasive technique to realize liquid biopsy of primary and metastatic HCC, which has become a research hotspot of early diagnosis and prognosis evaluation of HCC. In particular, they have not focused on the possibility of CTCs (including CTCs entering the blood after invading the portal vein branch) form vascular tumor thrombi from a single circulating tumor cell, and have not considered the possible leading role and molecular mechanism of CTCs as a vital component of MVI or PVTT in the formation of vascular tumor thrombi.

A variety of Inflammatory stimulating factors derived from HCC lead to the upregulation of cell adhesion molecules, combined with platelets, TF, stromal cells and various immune cells, and further promote the aggregation of CTCs in the portal vein and hepatic vein vascular endothelium, which facilitate the formation of vascular tumor thrombus. By analyzing the relevant literature, we speculate that circulating HCC cells could be binding carriers. High expression of TF on CTCs’ membrane initiates coagulation cascade reaction including combination with coagulation factors in the blood, the activation of platelets by thrombin and the local coagulation on the surface of HCC CTCs, eventually promoting the formation of CTM by continuous aggregation and adhesion of CTCs. Coagulation factors are mainly produced by the liver, so their concentrations in the liver is high. In addition to the pathological anatomy and hemodynamic changes caused by liver cirrhosis, HCC CTCs have the advantages of causing local coagulation in the liver, forming CTM and MVI, and homing implantation. MVI is mainly composed of cellular components such as HCC cells, platelets, CAFs, TAMs, neutrophils, and non-cellular components such as fibrin. These components constitute a special microenvironment suitable for HCC CTCs growth, resistance to anoikis and apoptosis, and escape immune attack. Moreover, they can provide the nutrition supply, promote blood vessel adhesion and transendothelial migration of HCC CTCs by secreting cytokines such as MMPs and VEGF.

This review proposes a mechanism of vascular tumor thrombus formation in HCC with CTCs as the core, which is conducive to a more comprehensive understanding of the pathological process of vascular invasion in HCC. Meanwhile it develops a new model of predicting MVI/PVTT based on HCC CTCs and its molecular classification, as well as combination therapy for relevant cells that facilitate MVI formation in the tumor microenvironment. MVI reflects the invasiveness of cancer cells. As the first step of tumor vascular invasion and metastasis, whether MVI has homology with CTCs derived from the primary focus is worth further study. The recurrence and metastasis of HCC involve complex and precise regulatory network, and the choice of clinical therapeutic strategies should also fully consider the key components in the tumor microenvironment. Targeted combination therapy may bring new hope to the cure of HCC in the future.
